# The Possible Association Between Chronic Toxoplasmosis and Type-2 Diabetes Mellitus In Women: A Case-Control Study

**DOI:** 10.7759/cureus.79120

**Published:** 2025-02-16

**Authors:** Khalil Mohammed

**Affiliations:** 1 Epidemiology and Medical Statistics, Umm Al-Qura University, Mecca, SAU

**Keywords:** chronic toxoplasmosis, diabetes mellitus, metabolic disorders, reproductive age, toxoplasma gondii

## Abstract

Introduction: The parasite *Toxoplasma gondii *is the source of toxoplasmosis. This infection can spread from mother to fetus during pregnancy, through contaminated food or water, or contact with infected cat feces. This study aims to investigate whether chronic toxoplasmosis is associated with an increased risk of type 2 diabetes mellitus (T2DM) in women.

Methods: A total of 274 samples were collected from women of reproductive age (18-55 years) as they were relevant for both chronic toxoplasmosis and T2DM. Serological testing was performed to detect the presence of *T. gondii* antibodies (IgM and IgG) to assess chronic infection, and fasting plasma glucose and HbA1c levels were measured for T2DM diagnosis.

Results: Among the participants, 46 (16.8%) were seropositive for chronic toxoplasmosis, and 68 (24.8%) were diagnosed with T2DM. Statistical analysis revealed that women with chronic toxoplasmosis were 2.3 times more likely to have T2DM compared to women without toxoplasmosis (OR = 2.289, CI: 1.171-4.473, p-value < 0.05). The study also found a significant association between education level and T2DM, with educated women being at lower risk of having T2DM (p-value < 0.05).

Conclusion: This study highlights a statistically significant association between chronic toxoplasmosis and T2DM. Women who were seropositive for chronic toxoplasmosis were more likely to have T2DM compared to seronegative individuals. These findings contribute to the growing body of evidence suggesting a potential link between chronic infections and metabolic disorders. Further research is needed to establish causation, elucidate underlying mechanisms, and explore potential interventions to mitigate the risk of T2DM in individuals with chronic toxoplasmosis.

## Introduction

Toxoplasmosis is an infection caused by the parasite *Toxoplasma gondii,* which can be transmitted through several routes, including contaminated food or water, exposure to infected cat feces, or in utero transmission from mother to fetus [1,2]. Chronic toxoplasmosis in pregnant women is a significant concern due to its potential impact on both the mother and the developing fetus. Chronic toxoplasmosis refers to a long-lasting infection that might persist for years. In some cases, individuals might stay asymptomatic, but the parasite can remain dormant in tissues, especially in the brains and muscles [3-5]. Chronic toxoplasmosis poses unique challenges in pregnancy, but with proactive management and education, risks can be mitigated. Pregnant women should be aware of the disease, its transmission routes, and preventive measures and should consult healthcare providers for personalized care and advice [6,7].

Type 2 diabetes mellitus (T2DM) is a chronic metabolic disorder characterized by insulin resistance and relative insulin deficiency. It is the most common form of diabetes, accounting for approximately 90% to 95% of all diabetes cases globally [8,9]. It results from a combination of genetic, environmental, and lifestyle factors that influence insulin function and secretion. Key contributors include obesity as a major risk factor, physical inactivity, sedentary lifestyles that can contribute to insulin resistance, and diet, particularly high consumption of sugary foods, refined carbohydrates, and saturated fats, which may increase the risk [10,11]. Common symptoms of T2DM include increased thirst, frequent urination, increased hunger, fatigue, blurred vision, slow-healing sores, frequent infections, areas of darkened skin, and unintended weight loss [12].

The association between chronic toxoplasmosis and T2DM is an emerging area of research that seeks to understand how infection by the parasite *T. gondii* may influence glucose metabolism and insulin resistance [13-15]. Chronic infection can manifest as asymptomatic or present mild, non-specific symptoms, but it can have long-term health implications, as chronic toxoplasmosis might influence glucose metabolism and contribute to the pathogenesis of T2DM [16].

Chronic toxoplasmosis can trigger inflammatory responses. Inflammation is known to contribute to insulin resistance, which is a core feature of T2DM. Elevated levels of pro-inflammatory cytokines may disrupt insulin signaling pathways [14,17]. Some studies suggest that infections can alter gut microbiota composition, which in turn affects metabolic processes. Dysbiosis has been linked to the development of insulin resistance and T2DM [18]. Some other studies suggest that toxoplasmosis may affect the production of hormones that regulate glucose metabolism. For instance, insulin and leptin levels could be influenced by the immune response to the parasite [19]. There is evidence to suggest that *T. gondii *can affect central nervous system functions, potentially influencing appetite regulation and energy expenditure, which could contribute to obesity and diabetes [20,21].

Epidemiological studies investigating the relationship between chronic toxoplasmosis and T2DM have produced mixed results. Some studies suggest a higher prevalence of toxoplasma infection in individuals with T2DM, indicating that chronic infection could be a contributing factor. However, other studies have not found a significant association, highlighting the complexity of the relationship and the need for further research to elucidate potential links [22]. While some evidence suggests a plausible link between chronic toxoplasmosis and increased susceptibility to T2DM through mechanisms involving inflammation and metabolic dysregulation, the relationship is not fully understood [14]. The present study was conducted to clarify if there is an association between chronic infection and T2DM and studies the effect of the duration of infection, age, and BMI on toxoplasmosis in women in Saudi Arabia.

## Materials and methods

Study design

This study comparatively analyzes women diagnosed with T2DM against a control group without diabetes to evaluate the prevalence of toxoplasmosis. It was conducted in Mecca and was approved by the Bioethical Committee at the Faculty of Public Health and Health Informatics, Umm Al-Qura University in Mecca, SAU (approval no. 435-1). All participants gave their informed consent for participation in the study.

The area was chosen based on its relevance to the study objectives, including the prevalence of *T. gondii* infection and the increasing burden of T2DM in the population between March 2023 and November 2024 as part of a toxoplasmosis research project on the women in the area. The inclusion criteria for participants were women of reproductive age, generally between 18 and 55 years, diagnosed with cases of T2DM confirmed via standardized criteria (e.g., fasting glucose levels, HbA1c) and confirmed chronic toxoplasmosis through serological testing (IgG antibodies), and women with no pregnancy at the time of the study and no history of other autoimmune, infectious diseases, or metabolic disorders that could confound results. Women with acute toxoplasmosis infections and pregnant women were excluded from the study.

Methods

An adequate sample size was drawn based on statistical power analyses considering the prevalence of both conditions in the population. The study involved a total of 274 samples collected from women of reproductive age (18 to 55 years). These samples were used to assess the presence of *T. gondii* antibodies (IgM and IgG) for diagnosing chronic toxoplasmosis and to measure fasting plasma glucose and HbA1c levels to evaluate T2DM. From these samples, about 96 (35%) were positive for chronic toxoplasmosis, or T2DM, or both, and 178 (65%) were used as controls. The method of sampling used in this study was convenience sampling. This sample size was considered adequate to provide statistically significant results and to explore the association between chronic toxoplasmosis and T2DM. Serological testing to determine the presence of *T. gondii* antibodies (IgM and IgG) assesses chronic infection and measures fasting plasma glucose and HbA1c. The study also collected demographic data (age, BMI, lifestyle factors), medical history, and current medications. We also included a comprehensive health questionnaire to assess some risk factors.

Diagnosis of chronic toxoplasmosis

The enzyme-linked immunosorbent assay (ELISA) method was used to diagnose chronic and acute toxoplasmosis in the serum samples. The test to diagnose human toxoplasmosis was conducted using the ELISA kit according to manufacturer procedures to detect specific IgM and IgG antibodies against *T. gondii*.

To do so, plates were pre-coated with *T. gondii-*specific antigens to capture the antibodies from the serum samples. Serum samples from participants were added to the wells. If antibodies (IgM or IgG) specific to *T. gondii *were present in the serum, they bound to the antigens on the plate. Unbound antibodies were washed away to reduce background noise and improve test specificity. A secondary, enzyme-linked antibody specific for human IgM or IgG was added to the wells. This antibody binds to the captured IgM or IgG from the serum. A substrate for the enzyme linked to the secondary antibody was added. The enzyme catalyzed a colorimetric reaction, resulting in the development of a measurable color. The intensity of the color was measured using a spectrophotometer. The absorbance readings correlated with the concentration of anti-toxoplasma antibodies in the serum.

High levels of IgG indicated either chronic infection or past exposure to *T. gondii*, meaning the individual had been infected in the past and developed immunity. The presence of IgM generally indicated a recent or acute infection with *T. gondii*. On the other hand, the diagnosis of T2DM was based on laboratory criteria: fasting plasma glucose (FPG) ≥ 126 mg/dL (7.0 mmol/L) after no caloric intake for at least eight hours.

Statistical analysis

The SPSS Statistics version 26 was used for data analysis. To summarize the basic features of the data, a chi-square test was conducted to determine if there was a significant association between two categorical variables. A p-value <0.05 was considered significant.

## Results

Effect of chronic toxoplasmosis on T2DM

A total of 274 women of reproductive age were investigated for the association between chronic toxoplasmosis and T2DM. Among the participants, 46 (16.8%) were seropositive for chronic toxoplasmosis, and 68 (24.8%) were diagnosed with T2DM. Statistical analysis revealed that women with chronic toxoplasmosis were 2.3 times more likely to have T2DM compared to women without toxoplasmosis (OR = 2.289, CI: 1.171-4.473, p-value < 0.05). The results suggest a statistically significant association between chronic toxoplasmosis and T2DM. Participants who were seropositive for chronic toxoplasmosis were more likely to have T2DM compared to those who were seronegative, as indicated by the OR and the p-value of <0.05 (Table 1). There was no significant relationship between chronic toxoplasmosis and obesity in the present study.

**Table 1:** The relationship between chronic toxoplasmosis, obesity, and T2DM The chi-square test was used to determine whether there is a significant association between the categorical variables. T2DM: Type 2 diabetes mellitus

Variable	Toxo Positive	Toxo Negative	Total	X^2^	P-value	95% CI	Odd Ratio
T2DM							
Yes	18(26.5%)	50(73.5%)	68	6.070	0.014	1.171-4.473	2.289
No	28(13.6%)	178(86.4%)	206				
Obesity							
Yes	58 (26.9%)	158 (73.1%)	216	2.263	0.132	0.837-3.711	1.762
No	10 (17.2 %)	48 (82.8%)	58				

No statistically significant association was observed between the age group and T2DM (p = 0.133). The odds of having T2DM were lower in the younger group (18 to 27 years) compared to those aged between 28 and 45 years, though this was not significant (OR = 0.583, 95% CI: 0.286-1.186). A statistically significant association was found between education level and T2DM (p= 0.013). Educated women were less likely to have T2DM than uneducated women (OR = 0.274, 95% CI: 0.092-0.812). No significant association was observed between working status and T2DM (p = 0.550). The odds of T2DM were slightly higher in working women than in nonworking women (OR = 1.276, 95% CI: 0.582-2.758). Although the association between abortion history and T2DM was not statistically significant (p = 0.065), women with a history of abortion were more likely to have T2DM (OR = 1.815, 95% CI: 0.958-3.436) (Table 2). These results highlight a significant association between education level and T2DM, with educated women being at lower risk. Other factors, such as age, occupation, and history of abortion, did not show statistically significant associations but may still suggest trends worth further exploration.

**Table 2:** Risk factors associated with T2DM The chi-square test was used to determine whether there is a significant association between the categorical variables. T2DM: Type 2 diabetes mellitus

Variable	Toxoplasmosis positive	Toxoplasmosis negative	Total	X^2^	p-value	95% CI	OR
Age (Years)							
18-27	12 (12.2%)	86 (87.8%)	98	2.250	0.133	0.286-1.186	0.583
28-45	34 (19.3%)	142 (80.7%)	176				
Education							
Educated	40 (15.4%)	219 (84.6%)	259	6.120	0.013	0.092-0.812	0.274
Uneducated	6 (40.0 %)	9 (60.0 %)	15				
Occupation							
Working	10 (19.6%)	41 (80.4%)	51	0.357	0.550	0.582-2.758	1.276
Nonworking	36 (16.1%)	187 (83.9%)	223				
Abortion							
Yes	23 (22.1%)	81 (77.9%)	104	3.450	0.065	0.958-3.436	1.815
No	23 (13.5%)	147 (86.5%)	170				

Risk factors associated with chronic toxoplasmosis

There was no statistically significant association between age group and chronic toxoplasmosis (p = 0.207). The odds of having chronic toxoplasmosis were lower in the younger group (18 to 27 years) compared to the older group (28 to 45 years), though not significant (OR = 0.684, 95% CI: 0.378-1.237). No significant association was observed between education level and chronic toxoplasmosis (p = 0.657). Educated women had slightly higher odds of chronic toxoplasmosis compared to uneducated women, though this was not significant (OR = 1.340, 95% CI: 0.376-4.898). No significant association was found between occupation and T2DM (p = 0.340). Working women had lower odds of chronic toxoplasmosis compared to nonworking women (OR = 0.694, 95% CI: 0.327-1.474). There was no statistically significant association between abortion history and chronic toxoplasmosis (p = 0.418). Women with a history of abortion had slightly lower odds of chronic toxoplasmosis compared to those without, though not significant (OR = 0.789, 95% CI: 0.444-1.402). No statistically significant associations were observed between chronic toxoplasmosis and the evaluated risk factors (age, education, occupation, and abortion). However, the results provide insights into possible trends that may warrant further investigation with a larger sample size (Table 3).

**Table 3:** Risk factors associated with chronic toxoplasmosis The chi-square test was used to determine whether there is a significant association between the categorical variables.

Variable	Toxoplasmosis positive	Toxoplasmosis negative	Total	X^2^	p-value	95% CI	OR
Age (years)							
18-27	20 (20.4%)	78 (79.6%)	98	1.590	0.207	0.378-1.237	0.684
28-45	48 (27.3%)	128 (72.7%)	176				
Education							
Educated	65 (25.1%)	194 (74.9%)	259	0.197	0.657	0.376-4.898	1.340
Uneducated	3 (20.0 %)	12 (80.0 %)	15				
Occupation							
Working	10 (19.6%)	41 (80.4%)	51	0.912	0.340	0.327-1.474	0.694
Nonworking	58 (26%)	165 (74.0%)	223				
Abortion							
Yes	23 (22.1%)	81 (77.9%)	104	0.656	0.418	0.444-1.402	0.789
No	45 (26.5%)	125 (73.5%)	170				

## Discussion

More comprehensive studies, including longitudinal research and clinical trials, are necessary to clarify the association, understand the underlying mechanisms, and explore whether treating chronic toxoplasmosis might have implications for diabetes management. Future research should focus on large-scale epidemiological studies, experimental models, and clinical trials to better understand the mechanisms at play [23]. Investigating potential therapeutic interventions targeting the parasite in individuals with chronic infection may also reveal insights into blood glucose management and T2DM prevention. While there are intriguing associations and mechanisms proposed linking chronic toxoplasmosis and T2DM, more research is needed to establish causal relationships and clinical implications [22].

The findings of this study reveal a statistically significant association between chronic toxoplasmosis and T2DM. Women who were seropositive for chronic toxoplasmosis were found to be at a greater risk of developing T2DM compared to seronegative women, as evidenced by the OR and the corresponding p-value [24]. This association indicates that chronic toxoplasmosis may act as a potential risk factor for T2DM or contribute to its pathogenesis [25,26]. The observed link between chronic toxoplasmosis and T2DM could be explained by several biological mechanisms. Chronic toxoplasmosis is caused by persistent infection with the parasite *T. gondii*, which is known to induce chronic low-grade inflammation. This inflammatory state can contribute to insulin resistance, a key mechanism underlying the development of T2DM [27]. Additionally, *T. gondii* infection may disrupt normal metabolic processes, including glucose homeostasis, by affecting cytokine production or impairing pancreatic beta-cell function, which could lead to T2DM [28].

The findings of this study are consistent with previous reports that suggest a link between chronic infections and metabolic diseases. Chronic infections, including toxoplasmosis, have been implicated as potential contributors to the development of non-communicable diseases such as T2DM. Studies investigating other persistent infections (e.g., cytomegalovirus or *Helicobacter pylori*) have also found similar associations with insulin resistance and diabetes. These findings highlight the potential role of infections in the etiology of metabolic disorders [14,29].

The association between chronic toxoplasmosis and T2DM has important public health and clinical implications. First, it underscores the need to consider infectious diseases as potential contributors to chronic conditions such as diabetes [26]. Second, it highlights the importance of early detection and management of chronic toxoplasmosis, particularly in populations at higher risk for T2DM [30]. Screening for toxoplasmosis seropositivity in individuals with prediabetes or metabolic syndrome may help identify those at risk of developing diabetes. Furthermore, preventive measures, such as health education about toxoplasmosis transmission and infection control, could reduce the burden of both chronic toxoplasmosis and its potential metabolic complications.

In conclusion, this study highlights a statistically significant association between chronic toxoplasmosis and T2DM. Women seropositive for chronic toxoplasmosis were more likely to have T2DM compared to seronegative individuals. These findings contribute to the growing body of evidence suggesting a potential link between chronic infections and metabolic disorders. Further research is warranted to establish causation, elucidate underlying mechanisms, and explore potential interventions to mitigate the risk of T2DM in individuals with chronic toxoplasmosis.

Limitations

There are several potential limitations that should be considered. The diagnosis of chronic toxoplasmosis was determined by serological tests that may result in a false positive or negative. Also, diagnosing T2DM was based on a single test, which may not capture all cases accurately.

## Conclusions

This study highlights a statistically significant association between chronic toxoplasmosis and T2DM. Women seropositive for chronic toxoplasmosis were more likely to have T2DM compared to seronegative individuals. These findings contribute to the growing body of evidence suggesting a potential link between chronic infections and metabolic disorders. Further research is warranted to establish causation, elucidate underlying mechanisms, and explore potential interventions to mitigate the risk of T2DM in individuals with chronic toxoplasmosis.
